# Depression, Anxiety, and Suicidal Ideation in Chinese University Students During the COVID-19 Pandemic

**DOI:** 10.3389/fpsyg.2021.669833

**Published:** 2021-08-05

**Authors:** Shuang-Jiang Zhou, Lei-Lei Wang, Meng Qi, Xing-Jie Yang, Lan Gao, Suo-Yuan Zhang, Li-Gang Zhang, Rui Yang, Jing-Xu Chen

**Affiliations:** ^1^Beijing HuiLongGuan Hospital, Peking University HuiLongGuan Clinical Medical School, Beijing, China; ^2^Department of Psychology, Chengde Medical University, Chengde, China; ^3^Beijing Anding Hospital, Capital Medical University, Beijing, China

**Keywords:** COVID-19, depressive symptoms, anxiety symptoms, suicidal ideation, social support

## Abstract

Coronavirus disease-2019 (COVID-19) pandemic has seriously threatened the global public health security and caused a series of mental health problem. Current research focuses mainly on mental health status and related factors in the COVID-19 pandemic among Chinese university students. Data from 11133 participants was obtained through an online survey. The Patient Health Question-9 (PHQ-9) was used to assess depressive symptoms, the Social Support Rate Scale (SSRS) was used to assess social support. We also used 7-item Generalized Anxiety Disorder Scale (GAD-7) to assess anxiety symptoms. Totally, 37.0% of the subjects were experiencing depressive symptoms, 24.9% anxiety symptoms, 20.9% comorbid depressive and anxiety symptoms, and 7.3% suicidal ideation. Multivariable logistic regression analysis revealed an increased presence of mental health problems in female students, graduate students, and those with personal COVID-19 exposure. Awareness of COVID-19, living with family were protective factors that reduced anxiety and depression symptoms. In addition, male, personal COVID-19 exposure, depressive and anxiety symptoms were risk factors for suicidal ideation. Social support, COVID-19 preventive and control measures, prediction of COVID-19 trends, living with family and graduate students are protective factors for reducing suicidal ideation.

## Introduction

Coronavirus disease-2019 (COVID-19) is an acute respiratory infection disease caused by severe respiratory syndrome coronavirus 2 (SARS-CoV-2). It is characterized by developing rapidly, widespread, and strong infectivity, and lack of specific treatment (Chan et al., 2020). The global COVID-19 epidemic is now nearly 1 year, with the coming of autumn and winter, COVID-19 is now worsening again in many countries. The COVID-19 epidemic has also caused many mental health problems (Bao et al., 2020). Since the COVID-19 outbreak, studies have shown that a high percentage of children, adolescents, and adults have psychological problems, such as suicidal tendencies, sleep disruption, anxiety, depression, and behavioral problems (Altena et al., 2020; Li et al., 2020; Liu et al., 2020; Purtle, 2020; Yang et al., 2020; Zhou et al., 2020). Some experts especially highlighted the urgency and importance of evaluating and managing mental health problems during the COVID-19 pandemic (Chenneville and Schwartz-Mette, 2020; Lai et al., 2020; Wang Y. et al., 2020). Social support is a resource in social relationships that may buffer or mitigate the effects of life events and other stressors (Kessler et al., 1985). Studies have shown that social support is associated with suicidal thoughts, anxiety, and depressive symptoms (Amit et al., 2020). High levels of social support can reduce suicidal ideation (Bi et al., 2020).

University students undergo a critical transition as they become independent and responsible for their own health during university years (Kim et al., 2018), and experience higher psychological stress levels (e.g., academic pressure, living conditions, financial situation) than their peers in the general population (Auerbach et al., 2018; Recabarren et al., 2019). University life is the peak period for the first onset of common mental disorders such as anxiety, depression and insomnia (Auerbach et al., 2018). This has brought a lot of troubles to university students, seriously affecting their social functions, study and life (Beiter et al., 2015; Scanlan and Hazelton, 2019; Jenkins et al., 2020). In addition, suicidal ideation are also common among university students (Lew et al., 2020). The anxiety and depression symptoms in college students are related to stress factors such as earthquakes, floods and epidemics (Huang et al., 2003; Li et al., 2003, 2011). University students’ mental health problems have increased significantly during the outbreak of infectious diseases such as influenza A (H1N1) (Kanadiya and Sallar, 2011; Main et al., 2011).

During the outbreak of COVID-19, university students’ education, including university studies and internship, was completed halted, which implies long hours at home and can lead to disordered rhythms of life and irregular sleep patterns. Moreover, the pandemic has brought the risk of infection and death. These may be traumatic experiences and have a psychological impact on this population. There have been no studies on anxiety, depressive symptoms, suicidal thoughts and social support among college students in China.

Since Chinese university students have been exposed to a persistent source of distress during the public health emergency, it is imperative to evaluate and respond to their mental health issues. But there have been no studies on anxiety, depressive symptoms, suicidal thoughts and social support among college students in China. For this purpose, the prevalence and potential factors contributing to depressive and anxiety symptoms, suicidal ideation, social supporting were detected.

## Materials and Methods

### Participants

This research was a cross-sectional study, students were invited to complete a battery of online questionnaires through the Wenjuanxing platform from March 1 to 15, 2020. Inclusion criteria were full-time university students, including undergraduate and graduate students, living in mainland China, equal to or greater than 18 years of age. Participants who failed to complete the questionnaire were excluded from the study. Students signed online informed consent before participating in the study. The study was also approved by the Ethics Committee of Beijing Huilongguan Hospital.

### Procedure

#### Sociodemographic Factors

Demographic information, including gender, region, grade, and whether living with family were collected.

#### Assessment of COVID-19 Exposure and Awareness of COVID-19

Individual COVID-19 exposure was defined as a person who has been diagnosed with COVID-19, or a person who has a history of close contact with a COVID-19 patient in a mandatory isolation or medical observation. We used a self-made questionnaire to investigate university students’ awareness of COVID-19. The questionnaire consisted of three main questions. The first question is about whether the subject is familiar with COVID-19. We asked the subject whether he/she has taken preventive and control measures to prevent COVID-19 infection for the second question. The final question asked the subject about his/her attitude toward the prediction of COVID-19 trends. The score for all the questions were ranged from 1 to 5.

#### Assessment of Depressive Symptoms

We used the Chinese version of the 9-item Patient Health Questionnaire (PHQ-9) to assess the severity depressive symptoms (Spitzer et al., 1999). The questionnaire consists of 9 items, For each item, the answer consists of four choices: Not at all, several days, more than a week, and almost every day. The corresponding score is 0, 1, 2, and 3. The symptom severity is determined by the total score, with 5–9 being mild, 10–14 being moderate, 15–19 being moderately severe, and 20–27 being severe.

#### Assessment of Anxiety Symptoms

We used Chinese version of the 7-item Generalized Anxiety Scale (GAD-7) to assess participants’ anxiety symptoms (Spitzer et al., 2006), with symptom prevalence on a scale from 0 (not at all) to 3 (nearly every day). The symptom severity is determined by the total score, with 5–9 being mild, 10–14 being moderate, and 15–21 being severe.

#### Assessment of Suicidal Ideation

Suicidal ideation among college students was assessed by single item (item 9) of PHQ-9, which asked participants how often they thought they would be better off dead. Suicidal ideation is divided into four grades: From 0 (not at all) to 3 (nearly every day). The higher the level, the more serious the suicidal ideation.

#### Assessment of Social Support

The social support scale developed by Xiao Shuiyuan was used to evaluate the social support of college students. There are 10 items in this scale, including subjective support, objective support and utilization of support. A higher score indicates a higher level of social support or utilization. Previous studies have shown that the social support scale has good reliability and validity (Shuiyuan, 1987).

### Data Analysis

We used SPSS 24.0 for data analysis. We use percentages to show the proportion of depression symptoms, anxiety symptoms, suicidal ideation. The chi-square test was used to analyze the categorical variables. Logistic regression was used to explore the predictors of depressive or anxiety symptoms, and suicidal ideation. The level of significance was set at *p* < 0.05 (two-sided).

We used the Process macro program in SPSS to conduct the mediation effect analysis. In the mediating effect model, whether there is COVID-19 exposure is an independent variable, suicide concept is a dependent variable, subjective support and objective support are mediators. The results of the mediation analysis are presented in the form of plots. We used bootstrap to test the mediating effect. The sample size was set to 5,000, and the 95% confidence interval of indirect effect did not include zero, indicating that the mediating effect was significant.

## Results

A total of 11,372 participants completed the online questionnaires. After removing those answering less than 3 min or living abroad, 11,133 participants (18–35 years old, median = 21) from 31 provincial-level regions of mainland China, except Macau and Hong Kong, were involved in the current study, giving a response prevalence of 97.9%. Table 1 shows that 62.3% of the participants were female, 56.4% were urban residents, 90.3% were graduate students, 95.5% were living with their families, and 7.2% had exposure to COVID-19.

**TABLE 1:** Socio-demographic characteristics and association with depressive and anxiety symptoms.

Variables	n	%	Depressive symptoms			Anxiety symptoms			Suicidal ideation		
			N	%	*P*	n	%	*P*	n	%	*p*
**Gender**					<0.001			<0.001			0.007
Male	4,195	37.7	1,424	33.9		956	22.8		341	8.1	
Female	6,938	62.3	2,695	38.8		1,811	26.1		469	6.8	
**Region**					0.165			0.866			0.080
Urban resident	6,284	56.4	2,360	37.6		1,558	24.8		481	7.7	
Rural resident	4,849	43.6	1,759	36.3		1,209	24.9		329	6.8	
**Grade**					0.003			<0.001			0.958
Undergraduates	10,053	90.3	3,674	36.5		2,477	24.3		731	7.3	
Graduate students	1,080	9.7	445	41.2		320	29.6		79	7.3	
**Living with family**					<0.001			<0.001			<0.001
Yes	10,628	95.5	3,850	36.2		2,552	24.0		711	6.7	
No	505	4.5	269	53.3		215	42.6		99	19.6	
**COVID-19 exposure**					<0.001			<0.001			<0.001
Yes	801	7.2	369	46.1		272	34.0		84	10.5	
No	10,332	82.8	3,750	36.3		2,495	24.1		726	7.0	
**Total**	11,133	100	4,119	37.0		2,767	24.9		810	7.3	

A total of 37.0% participants experienced mild to severe depressive symptoms, 24.9% experienced mild to severe anxiety symptoms, and the comorbidity prevalence of depressive and anxiety symptoms was 20.9%. Moreover, 7.3% of the students had suicidal ideation. The distribution of age among the three groups: With and without symptoms of depression, with and without symptoms of anxiety, with and without suicidal thoughts was non-normal (*P* < 0.001 for all Kolmogorov-Smirnova tests), so the Non-parametric Mann-Whitney test was used to compare the ages of all three groups. But only the group with anxiety symptoms was older than those without anxiety symptoms group (*P* < 0.001). As shown in Table 1, there were no differences in depressive and anxiety symptoms, suicidal ideation among university students between different regions. The proportion of depressive symptoms and anxiety symptoms among female students was higher than male students (38.9 vs. 33.9%; 26.1 vs. 22.8%). But the proportion of suicidal ideation for male students was higher than female students (8.1 vs. 6.8%). Depressive and anxiety symptoms were more likely to occur in graduate students than in undergraduates (41.2 vs. 36.5%; 29.6 vs. 24.3%), but there was no difference between undergraduate and graduate students for suicidal ideation. The differences in depressive and anxiety symptoms, suicidal ideation between students living with and without their families were statistically significant (53.3 vs. 36.2%; 42.6 vs. 24.0%; 19.6 vs. 6.7%). Students with COVID-19 exposure reported more depressive and anxiety symptoms, suicidal ideation than those without COVID-19 exposure (46.1 vs. 36.3%; 34.0 vs. 24.1%; 10.5 vs. 7.0%).

As shown in Table 2, The higher scores of COVID-19 awareness, preventive and control measures, and COVID-19 trend prediction scores, the lower proportion of anxiety symptoms, depression symptoms, and suicidal ideation.

**TABLE 2:** The relationship between COVID-19 awareness and depressive and anxiety symptoms.

Variables	n	%	Depressive symptoms			Anxiety symptoms			Suicidal ideation		
			n	%	*P*	n	%	*P*	n	%	*P*
**COVID-19 knowledge**					<0.001			<0.001			<0.001
Very unfamiliar	128	1.1	66	51.6		45	35.2		23	18.0	
Unfamiliar	1,606	14.4	674	42.0		440	27.4		132	8.2	
Medium level	4,713	42.3	1,836	39.0		1,203	25.5		361	7.7	
Familiar	3,777	33.9	1,290	34.2		908	24.0		226	6.0	
Very familiar	909	8.2	253	27.8		171	18.8		68	7.5	
**Preventive and control measures**					<0.001			<0.001			<0.001
Very inconsistent	202	1.8	68	33.7		53	26.2		22	10.9	
Inconsistent	462	4.1	196	42.4		462	27.1		42	9.1	
Neutral	899	8.1	409	45.5		304	33.8		103	11.5	
Consistent	5,909	53.1	2,300	38.9		2,536	26.0		449	7.6	
Very consistent	3,661	32.9	1,146	31.3		749	20.5		194	5.3	
**Projections of COVID-19 trend**					<0.001			<0.001			<0.001
Very pessimistic	99	0.9	49	49.5		42	42.4		19	19.2	
Pessimistic	734	6.6	397	54.1		306	41.7		101	13.8	
Neutral	2,660	23.9	1,162	43.7		823	30.9		266	10.0	
Optimistic	6,434	57.8	2,209	34.3		1,410	21.9		373	5.8	
Very optimistic	1,206	10.8	302	25.0		186	15.4		51	4.2	

Table 3 multivariable logistic regression showed that there was an increased presence of depressive and anxiety symptoms in female students (OR_D_ = 1.24, 95% CI: 1.14–1.34; OR_A_ = 1.21, 95% CI: 1.11–1.33), graduate students (OR_D_ = 1.14, 95% CI: 1.00–1.30; OR_A_ = 1.18, 95% CI: 1.02–1.346), and those with COVID-19 exposure (OR_D_ = 1.42, 95% CI: 1.22–1.65; OR_A_ = 1.51, 95% CI: 1.29–1.76). We found that college students living with their parents (OR_D_ = 0.52, 95% CI: 0.44–0.63; OR_A_ = 0.46, 95% CI: 0.38–0.55), being familiar with COVID-19 (OR_D_ = 0.85, 95% CI: 0.81–0.89; OR_A_ = 0.92, 95% CI: 0.87–0.97), actively taking preventive and control measures (OR_D_ = 0.88, 95% CI: 0.84–0.92; OR_A_ = 0.87, 95% CI: 0.83–0.91), and being optimistic about projections of COVID-19 trends (OR_D_ = 0.71, 95% CI: 0.67–0.74; OR_A_ = 0.67, 95% CI: 0.63–0.70) were protective factors for depressive symptoms and anxiety symptoms. As for suicidal ideation, multivariable logistic regression showed that depressive (OR = 10.62, 95% CI: 7.84–14.38) and anxiety symptoms (OR = 5.56, 95% CI: 4.53–6.81) were risk factors. And female students (OR = 0.72, 95% CI: 0.61–0.84), graduate students (OR = 0.74, 95% CI: 0.57–0.97), living with family (OR = 0.48, 95% CI: 0.37–0.64), preventive and control measures (OR = 0.86, 95% CI: 0.75–0.91), and projections of COVID-19 trends (OR = 0.83, 95% CI: 0.60–0.71).

**TABLE 3:** Sociodemographic characteristics and COVID-19 awareness correlates with depressive and anxiety symptoms.

Variables	Depressive symptoms			Anxiety symptoms			Suicidal ideation		
	OR	95%CI	*P*	OR	95%CI	*P*	OR	95%CI	*P*
**Gender**									
Male	1			1			1		
Female	1.24	1.14–1.34	<0.001	1.21	1.11–1.33	<0.001	0.72	0.61–0.84	<0.001
**Grade**									
Undergraduate	1			1			1		
Graduate	1.14	1.00–1.30	0.051	1.18	1.02–1.36	0.025	0.74	0.57–0.97	0.028
**Living with family**									
No	1			1			1		
Yes	0.52	0.44–0.63	<0.001	0.46	0.38–0.55	<0.001	0.48	0.37–0.64	<0.001
**COVID-19 exposure**									
No	1			1			1		
Yes	1.42	1.22–1.65	<0.001	1.51	1.29–1.76	<0.001	1.17	0.89–1.52	0.262
**Awareness of COVID-19**									
COVID-19 knowledge	0.85	0.81–0.89	<0.001	0.92	0.87–0.97	0.001	1.00	0.91–1.09	0.937
Preventive and control measures	0.88	0.84–0.92	<0.001	0.87	0.83–0.91	<0.001	0.86	0.75–0.91	0.001
Projections of COVID-19 trend	0.71	0.67–0.74	<0.001	0.67	0.63–0.70	<0.001	0.83	0.60–0.71	<0.001
**Depressive symptoms**									
No	–			–			1		
Yes	–			–			10.62	7.84–14.38	<0.001
**Anxiety symptoms**									
No	–			–			1		
Yes	–			–			5.56	4.53–6.81	<0.001

**FIGURE 1:**
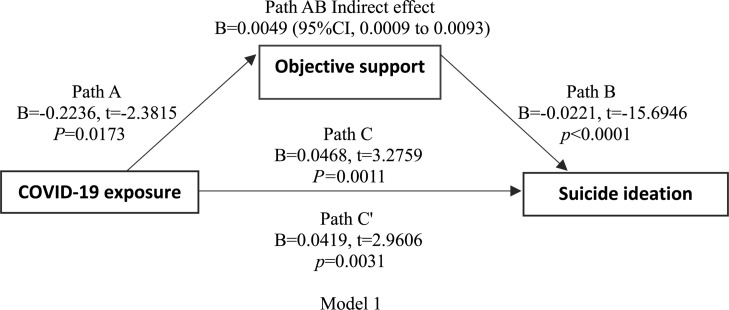
Model 1 shoes path diagram of the mediation model (X = COVID-19 exposure; Y = suicidal ideation). Path C represent the variance in COVID-19 exposure associated with suicidal ideation. Path C’ represent the association between COVID-19 exposure and suicidal ideation after taking into account objective support. Path AB is the mediation effect and is significant at *P* < 0.05.

**FIGURE 2:**
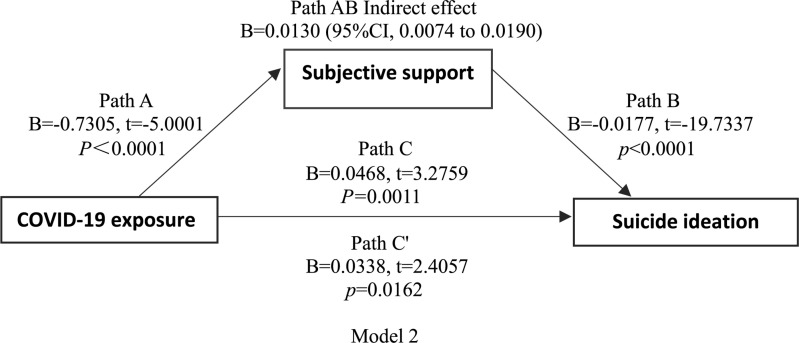
Model 2 shoes path diagram of the mediation model (X = COVID-19 exposure; Y = suicidal ideation). Path C represent the variance in COVID-19 exposure associated with suicidal ideation. Path C’ represent the association between COVID-19 exposure and suicidal ideation after taking into account subjective support. Path AB is the mediation effect and is significant at *P* < 0.05.

Linear regression showed that COVID-19 exposure was negatively correlated with subjective support, objective support, and suicidal thoughts. There was also a negative correlation between subjective support, objective support, and suicidal thoughts. Based on the regression results, we established a mediating effect model. As shown in Figures 1, 2 (models 1 and 2), the indirect effects between COVID-19 exposure and suicidal ideation through objective support, subjective support were significant, suggesting that models 1 and 2 were full mediation models.

## Discussion

Emotional problems are the most common psychological symptoms in university students (Auerbach et al., 2016), which may further increase during public health emergencies (Cao et al., 2020). This survey indicated the following main findings. Firstly, among university students in mainland China during the COVID-19 pandemic, 37.0% experienced depressive symptoms, 24.9% experienced anxiety symptoms, and 20.9% experienced comorbidity depressive and anxiety symptoms. Secondly, female gender, being a graduate, and personal COVID-19 exposure were independent risk factors and living with family was an independent protective factor for developing depressive and anxiety symptoms. Thirdly, awareness of COVID-19 is an important factor in reducing anxiety and depression symptoms, and suicide ideation.

In general, the prevalence of depressive and anxiety symptoms demonstrated in this study is clearly much higher than that in most previous studies during non-pandemic periods. For example, a meta-analysis, involving 39 studies with 32694 Chinese university students, indicated that the prevalence of depressive symptoms was 23.8% (95% CI: 19.9–28.5%) (Lei et al., 2016). Studies have shown that about 10% of undergraduate and graduate students report significant anxiety symptoms during their school years (Eisenberg et al., 2013; Auerbach et al., 2016). However, a relatively high prevalence of depressive and anxiety symptoms has also been observed in individual studies (Othman et al., 2019). On further analysis of the severity of mental health problems, it was found that mild depressive and anxiety symptoms were most common. In addition to anxiety and depression symptoms, college students’ suicidal ideation during the COVID-19 epidemic should also be concerned. Studies have shown that during the COVID-19 epidemic, the public has a high rate of suicidal ideation due to factors such as unemployment, home isolation, anxiety, depression, and insomnia symptoms (Bryan et al., 2020; Kawohl and Nordt, 2020; Li et al., 2020). But there have been no studies of college students. So it is worth mentioning that, even though only 7.3% students had suicide ideation, more attention should be paid to students with these characteristics.

There is now sufficient evidence to state that the female gender is a reliable risk factor for depressive and anxiety symptoms (Gater et al., 1998; Othman et al., 2019; Jenkins et al., 2020; Zhou et al., 2020). But our study found female students is a protect factor for suicidal ideation, which is consistent with previous research on factors influencing suicidal ideation among Chinese college students, it may be related to the great pressure placed on male college students by Chinese society (Lllhm, 2006). Graduate students, in contrast to undergraduate ones, have more negative emotions. This might be explained by more profound stresses regarding economic, marital, academic, interpersonal, and employment concerns as results of the pandemic. Although graduate students had more negative emotions, they had less suicidal ideation than undergraduates, this is not consistent with previous studies. Studies have shown that in the student population, for those older than 25 years old, the suicide rate of students is significantly higher than that of students younger than 25 years old. In the group of students aged 20–24, suicide rate of graduate students is higher than that of undergraduate students (Silverman et al., 1997; Hamilton and Schweitzer, 2000). In the present study, students living with family are related to lower risk of mental health problems, lower percentage of suicidal ideation. Some authors have demonstrated that family support, especially parental support, is very important and could effectively buffer the effects of high stress on anxiety symptoms and depressive symptoms, it also reduces suicidal ideation (Crockett et al., 2007; Gariepy et al., 2016; van Harmelen et al., 2016; Pruitt et al., 2020). Conversely, emotional loneliness caused by family disconnection is an important factor leading to mental health problems (Fernandez-Rouco et al., 2019). As predicted, COVID-19 exposure is closely related to bad moods. Individuals who were quarantined, irrespective of their wishes, suffered from isolation and directly faced the problems of infection, medical treatment, and even death (Elizarraras-Rivas et al., 2010; Oboho et al., 2015). But we also found that objective support, subjective support, was the intermediary between COVID-19 exposure and suicidal ideation. Previous studies have also shown that high levels of social support are protective factors for suicidal ideation (Hirsch and Barton, 2011; Parker et al., 2021). Therefore, providing social support to college students during the COVID-19 epidemic, especially for college students exposed to COVID-19, can reduce suicidal ideation.

Good awareness regarding infectious diseases may assist in the prevention of psychological problems (Khan et al., 2015). More accurate COVID-19 knowledge can reduce negative attitudes, potentially dangerous practices, fear and panic during the epidemic (Ren et al., 2020). Our findings supported this view and revealed COVID-19 awareness as an independent protective factor for mental health among university students. Of course, it is important to provide timely, specific and accurate health information about COVID-19 (Wang C. et al., 2020). Since the early stage of the COVID-19 pandemic, the Chinese government has provided essential COVID-19 knowledge to the public every day, through media campaigns via television, radio, WeChat, Tik Tok, and newspapers. However, it was found that only 42.1% students were familiar and 15.5% were unfamiliar with COVID-19 knowledge. Therefore, public health policy makers and health workers should attach importance to COVID-19 prevention training and health education for university students.

Based on the pandemic characteristics of COVID-19, the Chinese government and public authorities made efforts to facilitate the implementation of pandemic prevention measures. The practices were very cautious in the Chinese population: Decreased unnecessary outings, avoiding crowded places, wearing masks when going outside, and washing hands frequently (Zhong et al., 2020). Our study results were in agreement with a previous study, which suggested that precautionary measures could reduce the levels of anxiety and depression symptoms and psychological impact of the outbreak (Leung et al., 2003; Wang C. et al., 2020; Xiang et al., 2020).

During this survey period, the number of reported infection cases nationwide began to decline slightly, but the pandemic was spreading rapidly around the world and some imported cases occurred. Therefore, the public was urged to take more stringent preventive and control measures. Almost all students continued to stop their university studies and practice, and their range of activities was greatly restricted, which caused great inconvenience in their lives. Long-term self-isolation can make people bored and prone to focus too much on negative pandemic information, which also increases the risk of mental health problems (Gostic et al., 2020). However, our finding that the majority of students had an optimistic attitude about overcoming this crisis was unexpected. The most likely explanations for this situation are the openness and transparency of data, the effective and standardized implementation of preventive and control work in China (China, 2020). The optimistic attitude toward the prospects of COVID-19 could reduce depressive and anxiety symptoms, since risk perception has a greater correlation with mental health (Liao et al., 2014). However, recently, there has been a rebound of COVID-19 epidemic abroad, and many schools are facing another shutdown and class closure. The worsening COVID-19 epidemic may cause psychological problems among college students again. Therefore, our research also has certain guiding significance to alleviate the psychological problems of college students.

The key strengths of this study included the wide-ranging demographics and the largest sample studied to date. In addition, it was the first study to investigate the prevalence of anxiety, depression symptoms and suicidal ideation among university students and its influence during the COVID-19 epidemic. However, there are also some limitations to this study. First, the study adopted the method of convenience sampling to recruit subjects, which may lead to a lack of sample representativeness and an imbalance of the sample distribution. Second, we used the self-assessment questionnaire to assess the symptoms of anxiety and depression, so that reporting bias may exist when compared with the professional assessment. Third, all the data were collected in a cross-sectional survey, and therefore, causal relationships could not be established. Finally, the item 9 of PHQ-9 was mainly used for the evaluation of suicidal ideation. No professional questionnaire is used for the evaluation of suicidal ideation, which may not be systematic and detailed enough.

## Conclusion

In conclusion, the mental health status of university students has been affected during the COVID-19 pandemic, with a high prevalence of depressive symptoms, anxiety symptoms, and suicidal ideation. The female gender, graduates, living with family, personal COVID-19 exposure and awareness of COVID-19 were related factors for depressive and anxiety symptoms. In addition, our study showed that anxiety and depression symptoms are important risk factors for suicidal ideation. We also found that social support mediated between exposure and suicidal ideation. Providing adequate social support to university students may reduce suicide. While paying attention to the anxiety and depression symptoms of university students, we should also pay attention to the students’ suicidal ideation, and focus on the intervention of students with suicidal ideation.

## Data Availability Statement

The data that supports the findings of this study are available from the corresponding author upon reasonable request.

## Ethics Statement

The studies involving human participants were reviewed and approved by the Ethics Committee of Beijing HuiLongGuan Hospital. The patients/participants provided their written informed consent to participate in this study.

## Author Contributions

S-JZ and X-JY completed the design of the questionnaire. MQ was responsible for the examination of the contents of the questionnaire. LG, S-YZ, and L-GZ were responsible for the distribution and recovery of the questionnaire. L-LW and J-XC completed the statistical analyses. RY and J-XC received funding support for the research. S-JZ and MQ jointly completed the first draft of this manuscript. J-XC designed the whole study, provided guidance and reviewed and submitted the article. All authors have read and agreed with the published version of the manuscript.

## Conflict of Interest

The authors declare that the research was conducted in the absence of any commercial or financial relationships that could be construed as a potential conflict of interest.

## Publisher’s Note

All claims expressed in this article are solely those of the authors and do not necessarily represent those of their affiliated organizations, or those of the publisher, the editors and the reviewers. Any product that may be evaluated in this article, or claim that may be made by its manufacturer, is not guaranteed or endorsed by the publisher.
